# Impact of anemia on clinical outcomes in patients with acute heart failure: A systematic review and meta‐analysis

**DOI:** 10.1002/clc.24228

**Published:** 2024-01-29

**Authors:** Jiahui Pan, Meijun Liu, Jiamin Huang, Liuying Chen, Yizhou Xu

**Affiliations:** ^1^ Department of Cardiology, The Second Affiliated Hospital Zhejiang University School of Medicine Hangzhou Zhejiang People's Republic of China; ^2^ Department of Cardiology Hangzhou First People's Hospital Hangzhou Zhejiang People's Republic of China; ^3^ Department of the Fourth School of Clinical Medicine Zhejiang Chinese Medical University Hangzhou Zhejiang People's Republic of China

**Keywords:** acute heart failure, all‐cause heart failure events, all‐cause mortality, anemia

## Abstract

Anemia and acute heart failure (AHF) frequently coexist. Several published studies have investigated the association of anemia with all‐cause mortality and all‐cause heart failure events in AHF patients, but their findings remain controversial. This study is intended to evaluate the relationship between anemia and AHF. We systematically searched PubMed, Medline, the Cochrane Library, Embase, and Elsevier's ScienceDirect databases until July 30, 2023, and selected prospective or retrospective cohort studies to evaluate anemia for AHF. A total of nine trials involving 29 587 AHF patients were eventually included. Pooled analyses demonstrated anemia is associated with a higher risk of all‐cause heart failure event rate (OR: 1.82, 95% CI: 1.58−2.10, *p* < .01) and all‐cause mortality, both for short‐term (30 days) all‐cause mortality (OR: 1.91, 95% CI: 1.31−2.79, *p* < .01) and long‐term (1 year) all‐cause mortality (OR: 1.72, 95% CI: 1.27−2.32, *p* < .01). The evidence from this meta‐analysis suggested that anemia may be an independent risk factor for all‐cause mortality and all‐cause heart failure events in patients with AHF and might emphasize the importance of anemia correction before discharge.

## Graphical abstract

A total of nine trials involving 29 587 acute heart failure patients were eventually included. Pooled analyses demonstrated anemia is associated with a higher risk of all‐cause heart failure event rate (OR: 1.82, 95% CI: 1.58−2.10, *p* < .01) and all‐cause mortality, both for short‐term (30 days) all‐cause mortality (OR: 1.91, 95% CI: 1.31−2.79, *p* < .01) and long‐term (1 year) all‐cause mortality (OR: 1.72, 95% CI: 1.27−2.32, *p* < .01).

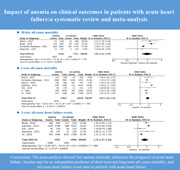

## 1 INTRODUCTION

Acute heart failure (AHF) is a prevalent kind of cardiovascular disease that poses a hazard to the health of 26 million individuals globally and has a high death rate and unfavorable prognosis.^1^ Even though several evidence‐based medications and device treatments have been developed during the past 20 years, the death and readmission rates for patients with AHF have not considerably decreased after discharge.^2^ Therefore, one of the biggest challenges facing cardiovascular medicine today is how to improve the prognosis of patients with AHF.

Anemia is defined by the World Health Organization (WHO) as Hb <13.0 g/dL in adult males and Hb <12.0 g/dL in adult women.^3^ The development of anemia in patients with AHF has been reported to be associated with a variety of factors, such as relative or absolute iron deficiency from various causes, decreased erythropoiesis due to abnormalities in the RAAS system, decreased erythropoietin due to chronic kidney disease, chronic disease anemia due to a proinflammatory state, and hemodilution, leading to varying degrees of ventricular remodeling and ultimately to increased mortality in patients with AHF.^4^, ^5^, ^6^, ^7^ Therefore, AHF and anemia frequently coexist and their comorbidity is associated with poor prognosis. According to a subgroup multiracial heart failure study, anemia was present in 1/3 to 1/2 of heart failure patients. Anemia was associated with a lower quality of life, a higher risk of morbidity and mortality, a higher rate of rehospitalization, and more treatment and management complications, with racial differences being significant.^8^ Furthermore, compared to non‐anemic patients, anemic patients are older, have higher levels of creatinine and natriuretic peptide, and are more likely to have other comorbidities.^9^

The prognostic value of anemia in AHF remains controversial, although a large body of data suggests a difference in the prognosis of patients with AHF between anemia and non‐anemia.^4^, ^10^ Amicis et al. reported anemia as an independent predictor of mortality in patients with AHF. In contrast, Li et al. showed that the effect of mild anemia on mortality then became nonsignificant after adjusting for confounders. Therefore, we performed this meta‐analysis to assess the prognostic role of anemia in all‐cause mortality and rehospitalization in patients with AHF.

## 2 METHODS

### 2.1 Search strategy

We systematically searched PubMed, Medline, the Cochrane Library, Embase, and Elsevier's ScienceDirect databases up to July 30, 2023, using relevant keywords and medical subject heading (MeSH) terms, including the following terms: acute heart failure, AHF, hemoglobin, Hb, anemia, hyphemia.

### 2.2 Study selection: Inclusion and exclusion criteria

Inclusion criteria for this study included: (1) Population: patients with the diagnosis of AHF; (2) predictors: baseline hemoglobin levels; (3) comparisons: anemia and non‐anemia patients; the diagnostic criteria for anemia were adopted from the WHO: Hb <13 g/dL for men and <12 g/dL for women; (4) outcomes: short‐term and long‐term all‐cause death or all‐cause heart failure events; (5) study design: prospective or retrospective cohort study; (6) data on risk estimates for all‐cause death or all‐cause rehospitalization associated with anemia were available. Exclusion criteria were as follows: (1) Studies that did not provide data on the estimated risk of death or hospitalization; (2) ambiguous study designs; (3) outcome measures of no interest; and (4) abstracts, case reports, case series studies, editorials, review articles, or non‐English language articles. All studies were independently screened and identified by two reviewers (J. P. and M. L.).

### 2.3 Quality assessment and data extraction

Two reviewers (L. C. and J. H.) independently collected data according to the above criteria, extracting information including first author, year of publication, nationality, number of patients, sex ratio, patient age, hemoglobin level, body mass index, comorbidities, vital signs, LVEF, NT‐proBNP, blood pressure, medication use, follow‐up time, and study outcome. We assessed the methodological quality of the included studies using the Newcastle−Ottawa scale (NOS). The scale judged studies on three dimensions: selection of study population, comparability of groups, and exposure or outcome of interest on a scale of 0−9, where studies with a NOS score of 7 or higher are considered high quality.^11^, ^12^, ^13^ When any disagreement arose during data extraction and quality assessment, the two reviewers reached a consensus through negotiation.

### 2.4 Data synthesis and analysis

All statistical analyses were performed by Jiahui Pan using Review Manager version 5.4 software (the Cochrane Collaboration 2014; Nordic Cochrane Center). The dominance ratio (OR) with a 95% confidence interval was used for dichotomous variables, and the mean difference was used for continuous variables. The heterogeneity of the studies was analyzed using the *I* ^2^ test.^14^ If the heterogeneity between studies was *I* ^2^ < 50%, *p* > .05, it indicated that the heterogeneity between studies was not statistically significant and the fixed‐effects model was used; if *I* ^2^ > 50%, *p* ≤ .05, it indicated that there was a statistically significant difference between studies and the random‐effects model was used to analyze the heterogeneity.^15^, ^16^ *p* < .05 was considered statistically significant.

## 3 RESULTS

### 3.1 Study selection

**Figure 1:**
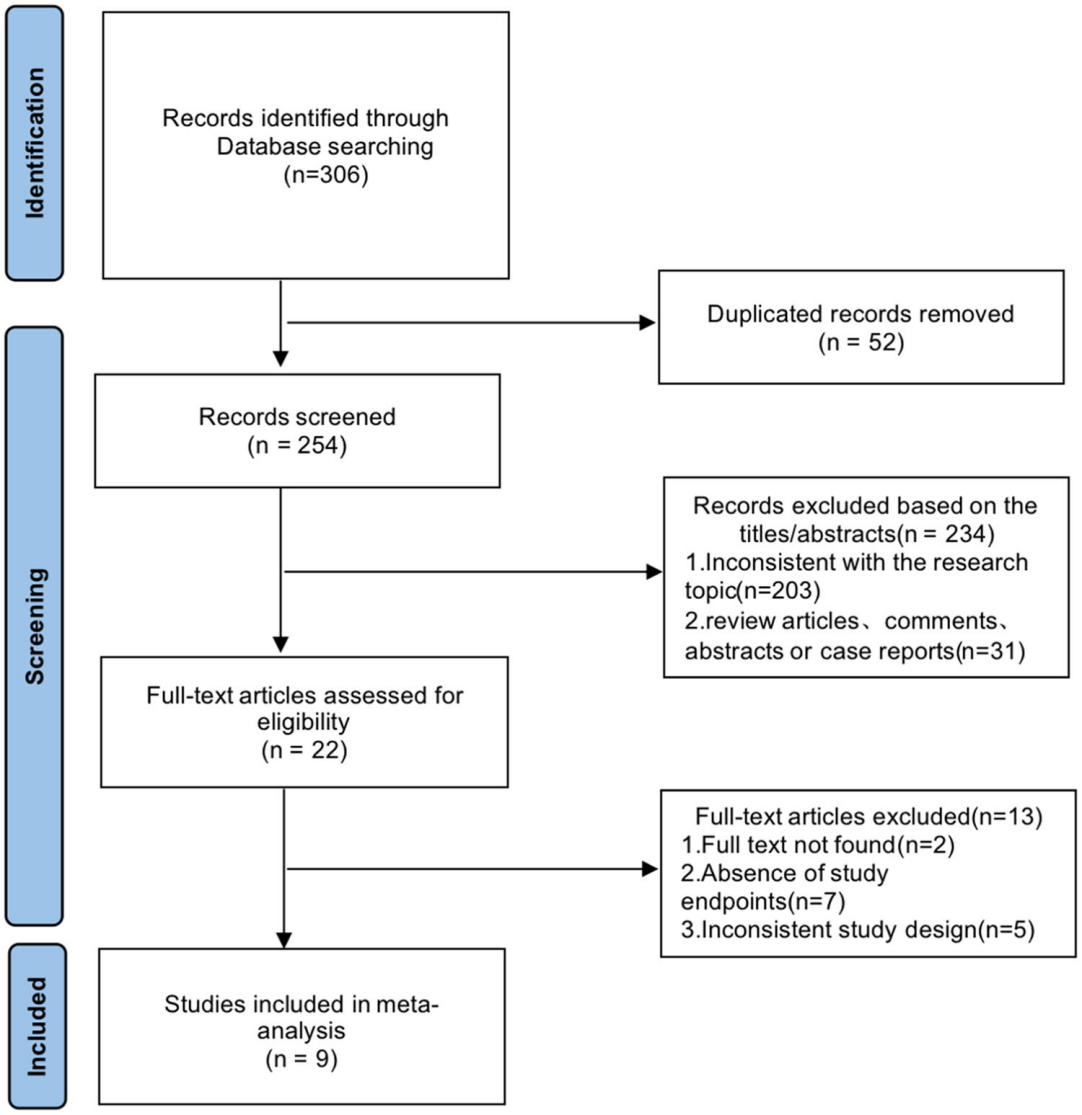
Flow diagram of study selection.

After the initial screening, we identified a total of 306 articles, 52 of which were duplicates. Browsing through the titles and abstracts revealed that 203 articles were excluded because they were inconsistent with the topic of the study, and another 31 articles were excluded because of the type of article such as review, commentary, abstract, case report, and so forth. The final detailed evaluation of the full text resulted in the inclusion of nine clinical trials including 29 587 patients.^9^, ^17^, ^18^, ^19^, ^20^, ^21^, ^22^, ^23^, ^24^ As shown in Figure 1.

### 3.2 Baseline characteristics and quality evaluation

Nine studies of 29 587 patients with AHF investigated prognostic differences associated with anemia. The mean age of study enrollees ranged from 63.1 ± 14.5 to 80.6 ± 9.5 years, with 53.7% women and the mean follow‐up period of 1−10 years. All studies were published from 2009 to 2022. Among the nine studies, five were addressed in Europe and the other four in Asia. For the endpoint events of the studies, the following three aspects were included: 30‐day all‐cause mortality, 1‐year all‐cause mortality, and 1‐year all‐cause heart failure events (readmission for AHF/all‐cause mortality + readmission for AHF/all‐cause mortality + heart transplantation + LVAD implantation). All nine included papers had NOS scores >7, indicating that the included studies were of high quality. Baseline information and quality assessment of the studies are detailed in Table 1.

**Table 1:** Baseline characteristics of included studies.

		Joerg et al.	Kajimoto et al.	Stojcevski et al.	Amicis et al.	Berge et al.	Kim et al.	Ye et al.	Fernández‐Rodríguez et al.	Li et al.
Country		Germany	Japan	Serbia	Italy	Netherlands	Korea	China	Spain	China
Publication year		2009	2014	2015	2017	2018	2019	2020	2021	2022
Patients (*n*)		627	4794	317	719	1769	384	3279	13454	4244
Female (*n*, %)		325 (51.8)	2013 (42.0)	150 (47.3)	399 (55.5)	644 (36.4)	191 (49.7)	1539 (46.9)	7473 (55.5)	2092 (49.3)
Age (year)	Anemia	74.6 ± 9.1	77.0 ± 11.9	76.7 ± 8.6	79.6 ± 8.9	63.1 ± 14.5	71.3 ± 11.8	74 (62−81)	80.6 ± 9.5	NR
	No anemia	72.7 ± 10.8	67.4 ± 14.3	72.2 ± 10.1	77.5 ± 10.6	64.1 ± 15.0	62.4 ± 16.4	67 (55−71)	79.2 ± 9.5	70 ± 12.1
Mean Hb (g/dL)	Anemia	11.0 ± 14.2	10.4 ± 1.6	10.7 ± 1.2	10.4 ± 1.4	NR	11.3 ± 1.8	10.7 (9.1−11.6)	NR	NR
	No anemia	14.3 ± 12.7	14.3 ± 1.9	14.0 ± 1.3	13.68 ± 1.2	NR	14.5 ± 1.5	14.1 (13.3−15.1)	NR	139 ± 12.7
BMI (kg/m^2^)	Anemia	NR	22.6 ± 4.3	NR	NR	24.8 ± 4.8	23.2 ± 5.1	23.7 (21.1−25.5)	NR	NR
	No anemia	NR	24.2 ± 4.9	NR	NR	25.4 ± 5.2	24.2 ± 3.7	23.7 (21.6−26.1)	NR	23.7 ± 3.9
Hypertension (%)	Anemia	NR	72.3	85.7	84.8	32.0	59.6	21.5	85.2	48.5
	No anemia	NR	65.5	75.7	81.7	34.0	54.4	13.8	81.4	47.0
Diabetes (%)	Anemia	NR	35.3	55.3	41.7	23.0	44.8	36.2	48.3	22.1
	No anemia	NR	31.8	41.7	31.7	20.0	18.4	25.0	34	18.0
Atrial fbrillation (%)	Anemia	NR	34.6	45.0	36.8	17.0	23.7	25.2	49.2	25.4
	No anemia	NR	37.8	56.5	34.1	26.0	36.8	31.4	48.4	27.1
Current smoke (%)	Anemia	NR	16.5	NR	15.4	NR	33.0	18.5	NR	6.7
	No anemia	NR	10.6	NR	15.2	NR	32.5	24.5	NR	9.5
HR (bpm)	Anemia	NR	92.2 ± 26.4	90.2 ± 24.2	NR	NR	88.1 ± 22.9	93 (80−110)	NR	NR
	No anemia	NR	107.2 ± 30.5	102.7 ± 29.5	NR	NR	91.2 ± 23.8	97 (82−116)	NR	90 ± 22.9
SBP (mmHg)	Anemia	127 ± 19	143.0 ± 34.9	NR	NR	NR	126.8 ± 23.4	135 (120−156)	140.3 ± 28.0	NR
	No anemia	131 ± 19	148.8 ± 38.7	NR	NR	NR	127.8 ± 24.9	130 (110−150)	142.7 ± 27.8	135 ± 24.4
DBP (mmHg)	Anemia	75 ± 10	78.2 ± 20.2	NR	NR	NR	78.6 ± 15.1	78 (68−88)	73.5 ± 17.4	NR
	No anemia	79 ± 10	88.5 ± 24.4	NR	NR	NR	80.2 ± 15.3	80 (70−90)	79.9 ± 17.4	NR
LVEF (%)	Anemia	43 ± 12	NR	41.1 ± 12.2	52.4 ± 13.6	NR	39 (29−51)	50 (38−60)	NR	NR
	No anemia	44 ± 12	NR	37.4 ± 13.5	45.8 ± 15.5	NR	37 (23−49)	40 (30−54)	NR	49.2 ± 14.7
Sodium (mmol/L)	Anemia	139 ± 4	139.1 ± 4.5	138 (135−140)	NR	136 ± 6	136.9 ± 4.0	138 (134−141)	NR	NR
	No anemia	140 ± 3	139.6 ± 4.2	139 (137−141)	NR	138 ± 5	138.0 ± 3.7	138 (135−141)	NR	139.4 ± 4.4
Scr (μmol/L)	Anemia	132 ± 89	1.64 ± 1.80	NR	125.6 ± 76.0	123 (94−200)	1.1 (0.8−1.6)	103.0 (75.2−177.4)	NR	NR
	No anemia	106 ± 50	1.13 ± 1.11	NR	96.9 ± 41.5	102 (82−130)	0.9 (0.7−1.2)	88.0 (69.9−105.8)	NR	NR
NT‐proBNP (pg/mL)	Anemia	NR	758 (380−1379)	5417 (2552−13 588)	NR	NR	5230 (2281−12 348)	5226 (2851−15 305)	NR	NR
	No anemia	NR	647 (330−1170)	2917 (1589−6725)	NR	NR	3731 (1542−7061)	4350 (2172−8041)	NR	2225 (70 85 960)
Diuretics (%)	Anemia	30	77.6	NR	NR	88.0	74.8	80.1	70.5	59.2
	No anemia	29	74.5	NR	NR	93.0	82.5	78.1	60.9	59.8
Beta‐blockers (%)	Anemia	<1	34.4	62.2	NR	15.0	72.9	22.4	41.1	49.1
	No anemia	<1	21.7	60.0	NR	19.0	78.9	38.7	39.3	53.2
ACEIs/ARBs (%)	Anemia	ACEIs: 34	ACEIs: 15.8 ARBs: 39.6	ACEIs: 59.0 ARBs: 67.8	NR	ACEIs: 49.0	69.1	20.9	ACEIs: 33.5 ARBs: 25.1	NR
	No anemia	ACEIs: 29	ACEIs: 13.0 ARBs: 28.7	ACEIs: 54.5 ARBs: 73	NR	ACEIs: 59.0	72.8	30.5	ACEIs: 33.7 ARBs: 24.4	NR
Digoxin (%)	Anemia	NR	12.5	NR	NR	NR	13.7	17.9	15.2	19.4
	No anemia	NR	13.2	NR	NR	NR	21.9	30.6	17.5	23.8
Nitrates (%)	Anemia	NR	NR	55.6	NR	35.0	27.4	23.5	20.6	NR
	No anemia	NR	NR	57.9	NR	37.0	16.7	28	14.6	NR
Follow		61 month	509 (383−775) day	12 month	18 month	10 year	528 day	372 (366−581) day	12 month	12.4 (11.9−12.6) month
NOS		9	8	7	8	9	8	8	8	8
All‐cause mortality at 30 days (*n*, %)	Anemia	NR	NR	11 (5.8)	51 (11.9)	170 (20.0)	NR	NR	820 (10.7)	NR
	No anemia	NR	NR	5 (4.4)	16 (5.5)	83 (9.0)	NR	NR	434 (7.5)	NR
All‐cause mortality at 1 years (*n*, %)	Anemia	126 (69.2)	870 (31.3)	NR	133 (31.0)	NR	48 (17.8)	572 (38.4)	2406 (31.4)	500 (24.5)
	No anemia	261 (58.7)	312 (15.5)	NR	55 (19.0)	NR	12 (10.5)	487 (27.2)	1228 (21.2)	367 (16.6)
All‐cause HF‐events at 1 years (*n*, %)	Anemia	NR	1387 (49.9)	50 (25.6)	NR	366 (43.0)	95 (35.2)	942 (63.2)	NR	NR
	No anemia	NR	608 (30.2)	17 (14.1)	NR	257 (28.0)	36 (31.6)	1014 (56.7)	NR	NR

Abbreviations: ACEI, angiotensin‐converting enzyme inhibition; ARB, angiotensin receptor blocker; BMI, body mass index; DBP, diastolic blood pressure; Hb, hemoglobin; HF, heart failure; HR, heart rate; LVEF, left ventricular ejection fraction; NOS, Newcastle−Ottawa scale; NR, not recorded; SBP, systolic blood pressure; Scr, serum creatinine.

### 3.3 Thirty‐day all‐cause mortality

**Figure 2:**
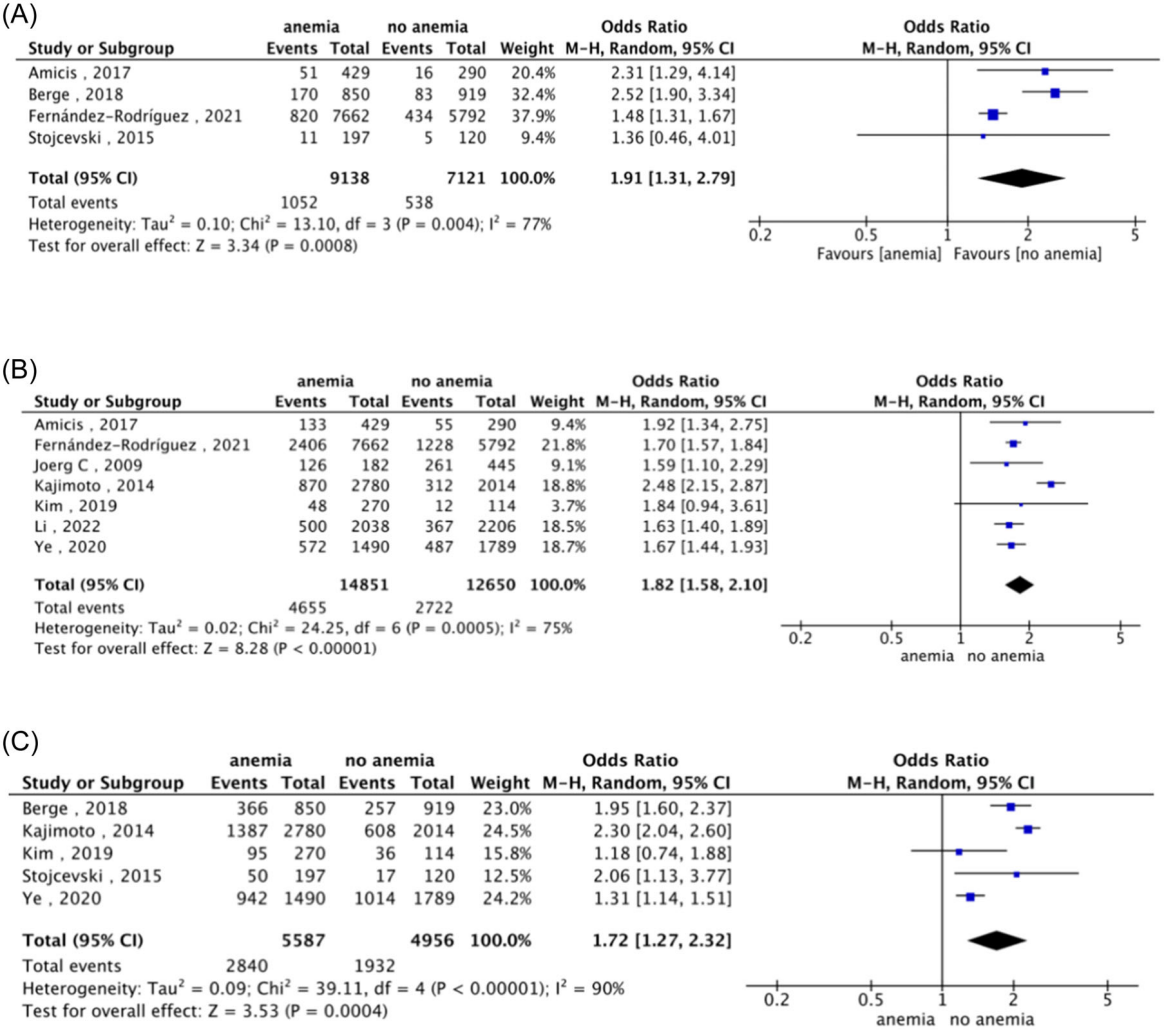
Effect of anemia on prognosis. (A) Association between anemia and 30‐day all‐cause mortality. (B) Association between anemia and 1‐year all‐cause mortality. (C) Association between anemia and 1‐year all‐cause heart failure events.

Four studies reported the incidence of 30‐day all‐cause mortality. As shown in Figure 2A, patients with anemia had a higher risk of all‐cause death 30 days after discharge compared with non‐anemic patients (OR: 1.91, 95% CI: 1.31−2.79, *p* < .01).

### 3.4 One‐year all‐cause mortality

Seven studies had an endpoint event of 1‐year all‐cause death. The combined analysis showed that AHF patients with anemia had a higher risk of combined all‐cause mortality than non‐anemic patients (OR: 1.82, 95% CI: 1.58−2.10, *p* < .01) (Figure 2B).

### 3.5 One‐year all‐cause heart failure events

Five studies evaluated 1‐year all‐cause heart failure event outcomes. Compared to non‐anemic patients, this study illustrates that anemia was linked to a greater incidence of all‐cause heart failure events in Figure 2C (OR: 1.72, 95% CI: 1.27−2.32, *p* < .01).

### 3.6 Heterogeneity analysis and subgroup analysis

**Figure 3:**
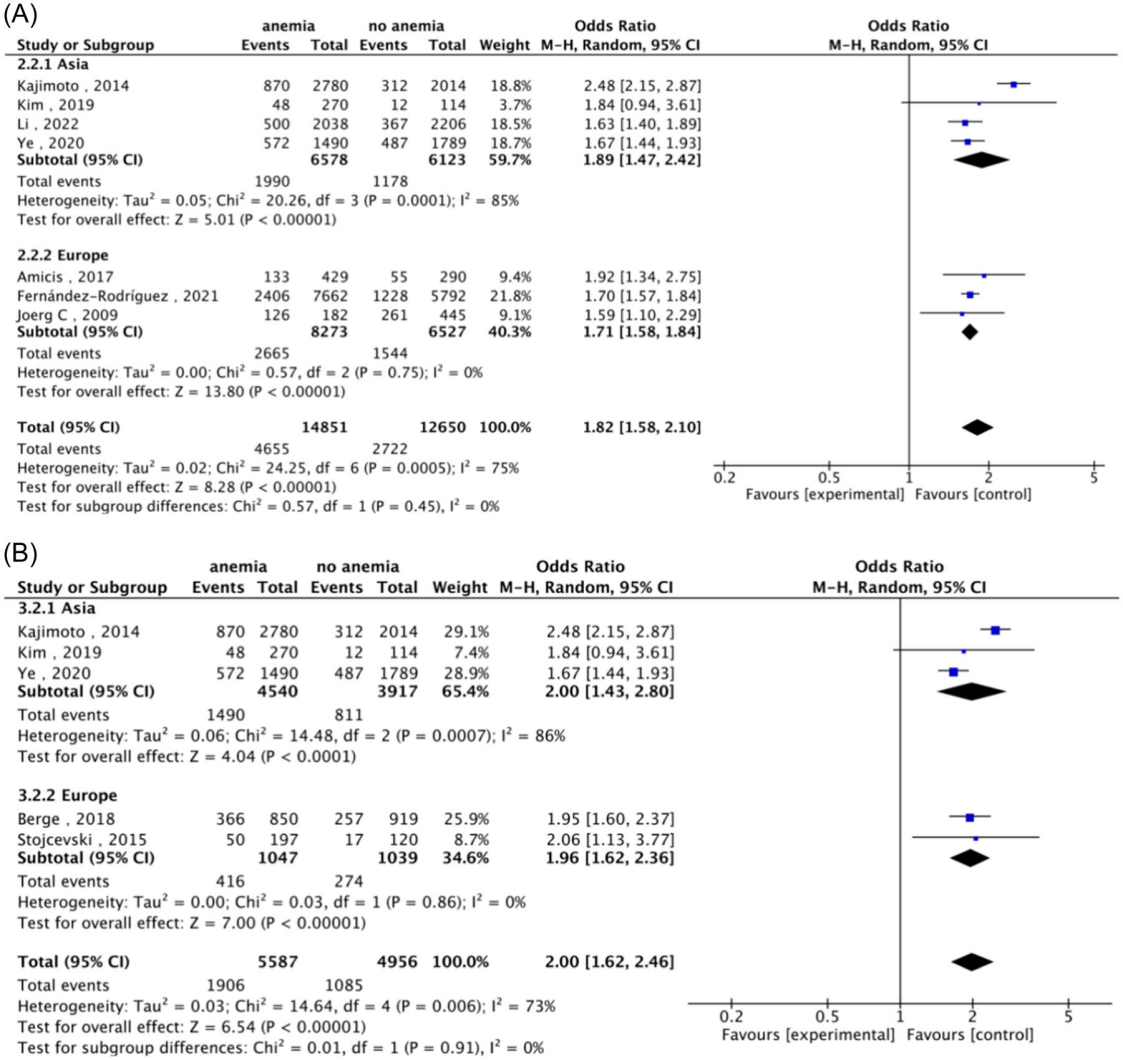
Subgroup analyses by ethnicity. (A) One‐year all‐cause mortality. (B) One‐year all‐cause heart failure events.

We found significant heterogeneity between studies included in the meta‐analysis in terms of 1‐year all‐cause mortality and all‐cause heart failure event rates (*I* ^2^ = 75%/90%). Therefore, subgroup analyses were performed based on race to identify sources of heterogeneity observed across studies. Results of the subgroup analysis showed that for all‐cause mortality and all‐cause heart failure events, heterogeneity was significantly lower in the European population (*I* ^2^ = 0%/0%); whereas this heterogeneity remained significant in the Asian population (*I* ^2^ = 85%/86%) (Figure 3).

### 3.7 Publication bias and sensitivity analysis

Due to the limited number of included studies less than 10, publication bias was not assessed in this meta‐analysis.

## 4 DISCUSSION

Based on a large amount of clinical and basic research data, patients with heart failure are more likely to have combined anemia, of which the most important pathophysiological mechanisms are iron deficiency and insufficient erythropoietin synthesis.^25^ The reasons for the increased incidence of anemia in heart failure patients are related to the following^5^, ^25^, ^26^, ^27^, ^28^, ^29^, ^30^: (1) Inadequate food intake and hypothalamic appetite regulation dysfunction lead to reduced iron intake; (2) irritation of the gastrointestinal mucosa after treatment with antiplatelet and or anticoagulant drugs, resulting in microscopic blood flow loss in the gastrointestinal system. At the same time, edema of the intestinal mucosa in the late stage of heart failure inhibits iron absorption; (3) plasma levels of inflammatory factors such as tumor necrosis factor‐alpha (TNF‐α) and interleukin‐6 (IL‐6) are elevated in patients with heart failure. IL‐6 stimulates hepcidin protein synthesis, which inhibits membrane transporter protein‐1 in the gastrointestinal tract, hepatocytes, and macrophages, ultimately reducing iron absorption in the gastrointestinal tract; (4) inflammatory factors TNF‐α and IL‐6 directly inhibit the proliferation and differentiation of erythroid progenitor cells in the bone marrow, and inhibits renal erythropoietin production by activating the transcription factor GATA‐binding protein 2 and the nuclear factor κ light chain enhancer of activated B cells; (5) hemodilution.

Furthermore, anemia is associated with poor prognosis in patients with heart failure. As early as 1995, scientists identified hypoproteinemia as a potential independent risk factor for the development and recurrence of congestive heart failure.^31^ A meta‐analysis including 20 studies with 97 699 patients with chronic heart failure demonstrated that all‐cause mortality and risk of rehospitalization in patients with chronic congestive heart failure were linked to the severity of anemia and hemoglobin levels.^32^ Another large meta‐analysis also confirmed a significant increase in mortality in patients with heart failure combined with anemia compared with non‐anemic patients.^33^

Patients with AHF are more likely to have anemia in combination, but the impact of anemia on the prognostic outcome of AHF remains controversial.^9^ Amicis et al.^17^ indicated that anemia was an independent predictor of death within 1 year in patients with AHF. Similarly, Stojcevski et al.^23^ demonstrated that low levels of hemoglobin at discharge were dramatically correlated with rehospitalization rates at 1, 6, and 12 months after discharge, and that each 1 g/L decrease in hemoglobin was associated with a 3.3% increase in rehospitalization rates at 1 year of follow‐up. Recently, however, Li et al.^22^ found that moderate‐to‐severe anemia (hemoglobin <11.0 g/dL) was independently related to mortality in AHF, but mild anemia did not independently predict patient death. Even Kim et al.^18^ concluded that anemia was not significantly associated with patient rehospitalization rates. To clarify whether anemia mediates the progression of AHF or whether it is an independent predictor of AHF severity and poor prognosis, we used meta‐analysis to determine the true conclusion. Pooled analysis showed that anemia was associated with a markedly increased risk of short‐term (30 days) all‐cause death, long‐term (1 year) all‐cause death, and long‐term (1 year) heart failure events after hospital discharge in patients with AHF.

Anemia contributes to poor outcomes in patients with AHF through multiple mechanisms. Patients with anemia have reduced oxygen delivery to metabolic tissues, giving rise to a range of hemodynamic and non‐hemodynamic compensatory mechanisms. The rapid and reversible non‐hemodynamic mechanism is an increase in erythrocyte 2,3‐diphosphoglycerate content, which shifts the oxygen dissociation curve to the right and decreases the affinity of hemoglobin for oxygen, resulting in elevated oxygen levels in tissues.^5^ The associated hemodynamic mechanisms are slower and more complex and produce adverse effects. The reduced number of circulating red blood cells results in a decrease in whole blood viscosity leading to a decrease in systemic peripheral vascular resistance, while low hemoglobin further activates nitric oxide‐mediated vasodilation leading to hypotension, which reflexively stimulates pressure receptors to activate pressor hormones to maintain peripheral tissue perfusion.^5^, ^30^, ^34^, ^35^ The heart is chronically overworked, which eventually leads to ventricular hypertrophy and myocardial remodeling.

However, patients with anemia are older and often have a combination of CKD, hypoproteinemia, diabetes mellitus, and so on, all of which have varying degrees of impact on prognosis.^36^, ^37^ Previous research has demonstrated that patients with AHF frequently have a combined iron deficit, that absolute ID usually persists, and that absolute ID increases the incidence of early rehospitalization in patients with AHF.^38^, ^39^ Ferric carboxymaltose can reduce the risk of rehospitalization, but the original studies did not information about the patients' iron deficiency status and treatments targeting anemia and iron deficiency, so it is not possible to determine the effect of iron deficiency status on the prognosis of AHF.^40^ What's more, heart failure drugs can potentially affect prognosis. In the EMPA‐REG trial, empagliflozin has been shown to increase hematocrit and hemoglobin to ameliorate anemia while lowering cardiovascular mortality, all‐cause mortality, and heart failure rehospitalization.^41^, ^42^ The mechanism might be that empagliflozin decreases hepcidin, improves bone marrow hematopoiesis, mitochondrial function, and muscle energetics, and then improves LVEF and peak VO_2_, affecting the prognosis of heart failure patients.^43^, ^44^, ^45^ Renin‐angiotensin system inhibitors have been verified through meta‐analysis to raise the risk of anemia. They cause decreased hemoglobin by preventing the synthesis of erythropoietin.^46^ They cause decreased hemoglobin by preventing the synthesis of erythropoietin.^47^ The lesser inhibitory effect of sacubitril/valsartan on hemoglobin compared to renin‐angiotensin system inhibitors was associated with a certain degree of induction of erythropoiesis by decreasing serum iron and hepcidin levels and increasing iron utilization with sacubitril/valsartan.^48^ More studies are needed to clarify whether the above factors independently of anemia predict prognosis in patients with AHF.

Although several meta‐analyses have explored the prognostic value of anemia on adverse outcomes in CHF, this study addresses the impact of anemia on the prognosis of AHF and incorporates the most recent relevant studies. Based on the results of this study, we believe that early identification and treatment of anemia is necessary for patients with AHF. In addition, comprehensive management of patients with AHF needs to be enhanced, and anti‐heart failure drugs need to be used rationally to further improve the clinical outcomes of patients.

Our meta‐analysis had the following limitations: first, the small number of studies included in this meta‐analysis, only nine non‐randomized observational studies with different follow‐up times, may have influenced the results; second, previous studies reported a greater prognostic impact of persistent anemia than transient anemia, but the included studies did not indicate whether hemoglobin levels were monitored on a continuous basis.^49^ And most of the included studies provide information on anemia at admission but not at discharge in patients with AHF, making it impossible to assess the predictive value of anemia at discharge for prognosis; third, we did not assess the effect of hemoglobin levels on mortality using continuous variable analysis; finally, there was a large heterogeneity among studies, and we performed subgroup analysis according to different ethnic groups to explore the sources of heterogeneity. The results of the subgroup analysis showed that significant heterogeneity remained in the Asian population and was significantly lower in the European population. Thus, ethnicity may be a source of heterogeneity. However, the information available was limited, so it was not possible to clarify whether the heterogeneity between studies came from other confounding factors such as gender, age, comorbidities, anemia etiology, anemia treatment, follow‐up time, and study design.

## 5 CONCLUSIONS

The meta‐analysis showed that anemia markedly influences the prognosis of AHF. Anemia may be an independent predictor of short‐term and long‐term all‐cause mortality, and all‐cause heart failure event rates in patients with AHF.

## CONFLICT OF INTEREST STATEMENT

The authors declare no conflict of interest.

## Data Availability

The data that support the findings of this study are available from the corresponding author upon reasonable request.
